# Effect of serum lactate dehydrogenase-to-albumin ratio (LAR) on the short-term outcomes and long-term prognosis of colorectal cancer after radical surgery

**DOI:** 10.1186/s12885-023-11446-5

**Published:** 2023-09-28

**Authors:** Xin-Peng Shu, Ying-Chun Xiang, Fei Liu, Yong Cheng, Wei Zhang, Dong Peng

**Affiliations:** Department of Gastrointestinal Surgery, The First Affiliated Hospital of Chongqing Medical University, Chongqing, 400016 China

**Keywords:** Colorectal cancer, Lactate dehydrogenase-to-albumin ratio, Short-term outcomes, Prognosis

## Abstract

**Background:**

Whether serum lactate dehydrogenase-to-albumin ratio (LAR) influenced the outcomes of colorectal cancer (CRC) patients after radical surgery remained unclear. Therefore, this study sought to examine how LAR influences the short-term and long-term outcomes of CRC patients who have undergone radical surgery.

**Methods:**

This study retrospectively included CRC patients who underwent radical resection between January 2011 and January 2020. We compared short-term outcomes, as well as overall survival (OS) and disease-free survival (DFS), among various groups. Both univariate and multivariate logistic regression analyses were utilized to pinpoint independent risk factors associated with overall complications and major complications. Moreover, Cox regression analysis were conducted for OS and DFS. Odds ratio (OR) and Hazard ratio (HR) were adjusted.

**Results:**

This study encompassed a cohort of 3868 patients. 3440 patients were in the low LAR group and 428 patients constituted the high LAR group. In the high LAR group, patients experienced significantly longer operative times (p < 0.01), larger intraoperative blood loss (p < 0.01), and extended postoperative hospital stays (p < 0.01). Additionally, the incidence of both overall complications (p < 0.01) and major complications (p < 0.01) was higher in the high LAR group compared to the low LAR group. Furthermore, LAR was emerged as an independent prognostic factor for overall complications [OR/95% CI: (1.555/1.237 to 1.954), p < 0.01] and major complications [OR/95% CI: (2.178/1.279 to 3.707), p < 0.01]. As for long-term survival, the high LAR group had worse OS in stage II (p < 0.01) and stage III (p < 0.01). In both stage II (p < 0.01) and stage III (p < 0.01), the high LAR group exhibited poorer DFS. Additionally, according to Cox regression analysis, LAR was identified as an independent predictor for both OS [HR/95% CI: (1.930/1.554 to 2.398), p < 0.01] and DFS [HR/95% CI: (1.750/1.427 to 2.146), p < 0.01].

**Conclusion:**

LAR emerged as an independent predictor not only for overall complications and major complications but also for both OS and DFS, highlighting its significance and deserving the attention of surgeons.

## Introduction

Globally, colorectal cancer (CRC) ranked as the third most common malignant tumor and the second leading cause of cancer-related fatalities. [1–3]. Although the surgical procedures and neoadjuvant chemoradiotherapies had become more and more mature, the prognosis of CRC was still not optimistic [4, 5]. It was reported that there were many biomarkers affecting prognosis in CRC patients, including lactate dehydrogenase (LDH), albumin [6].

On behalf of the key enzymes in the glycolytic path-way, LDH can convert pyruvate to lactate to support tumor cells [7, 8]. Some studies already revealed that high level of LDH was closely correlated with poor prognosis in many kinds of tumors, including lung cancer, pancreatic cancer, esophageal cancer and CRC [9–13]. On the other hand, the serum albumin level could serve as an indicator of the body’s the nutritional status. Previous studies had shown that abnormal albumin levels were correlated with poor prognosis of gastric cancer, and CRC [14, 15]. Therefore, elevated LDH and decreased albumin were indicators of poor survival.

The lactate dehydrogenase-to-albumin ratio (LAR), combining LDH and albumin, might be more effective than each alone. Some studies demonstrated that high LAR was associated with poor survival in cases of esophageal cancer, pancreatic cancer, hepatocellular carcinoma and gastric cancer [16–18]. There were few studies on the relationship between LAR and CRC [19, 20]. However, none of them did a comparison for the short-term outcomes including overall complications and major complications in different LAR groups, and none of them assessed OS and DFS across various tumor stages.

For this reason, this current study sought to examine the impact of LAR on the short-term outcomes and long-term prognosis of CRC patients who underwent radical surgery.

## Materials and methods

### Patients

**Fig. 1:**
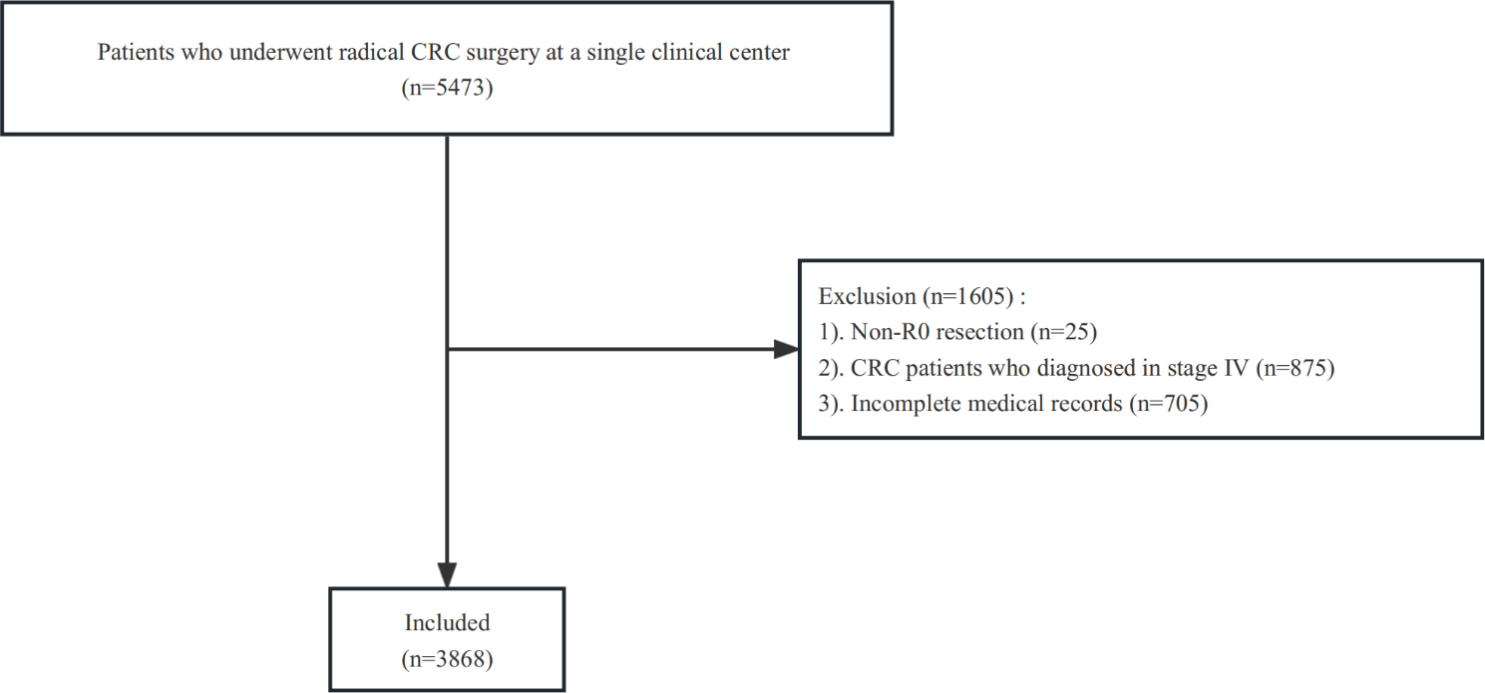
Flow chart of patient selection

Patients with CRC who underwent radical surgery at a single teaching hospital between Jan 2011 and Jan 2020 were included in this retrospective analysis. This study was conducted in accordance with the principles of the Helsinki Declaration. Moreover, the study received approval from the ethical committee at our institution (The First Affiliated Hospital of Chongqing Medical University, 2022-135-2), and the informed consent forms were acquired from all participantsAt the outset, a total of 5473 CRC patients who underwent radical resection were included in the study (n = 5473) Exclusion criteria encompassed: 1, non-R0 resection (n = 25); 2, CRC patients who diagnosed in stage IV (n = 875); and 3, incomplete medical records (n = 705). In total, 3868 patients were involved in the final analysis. (Fig. 1).

## Data collection

The baseline characteristics encompassed age, sex, body mass index (BMI), smoking, drinking, hypertension, type 2 diabetes mellitus (T2DM), neoadjuvant therapy, LDH, albumin, hemoglobin, LAR, tumor location, tumor node metastasis (TNM) stage, and tumor size. Short-term outcomes were assessed based on operation time, intraoperative blood loss, postoperative hospital stays, overall complications, and major complications. Long-term prognosis was evaluated through OS and DFS. Data collection involved retrieving information from the medical record system and conducting telephone interviews.

### Definitions

We determined the TNM stage based on the 8th Edition of the AJCC [21]. Complications were assessed using the Clavien-Dindo classification, with major complications defined as those graded ≥ III [22]. OS was calculated by the time from surgery to the last follow-up, or death from any cause. DFS was defined as the time from initiation of radical surgery to tumor recurrence or death.

### Optimal cut-off value and groups

The optimal cut-off value of 12.3 was identified using X-tile software. Then, patients with LAR ≤ 12.3 were classified into the low LAR group, while those with LAR values exceeding 12.3 were assigned to the high LAR group.

### Statistics

The statistical software SPSS (version 22.0) was utilized for data analysis. We represented continuous variables as mean ± standard deviation (SD) ,and categorical variables were expressed as n (%). Independent sample t-test and the Chi-square test or Fisher’s exact test were adopted to compare the difference for continuous variables and categorical variables, respectively. We assessed OS and DFS using the Kaplan-Meier method for stage I-III patients, comparing the two different groups. We conducted univariate and multivariate analyses to pinpoint predictors of overall complications and major complications, while Cox regression analyses were utilized to identify predictors of both OS and DFS. And we deemed a two-tailed p-value of less than 0.05 to be statistically significant.

## Results

### Patients

In this study, a total of 3868 patients were included. Based on the LAR cut-off value, 3440 patients were categorized into the low LAR group, while 428 patients comprised the high LAR group. Patients in the high LAR group had an older age (p < 0.01), a higher portion of females (p < 0.01), lower BMI (p < 0.01), smoking (p < 0.01), drinking (p < 0.01), hypertension (p = 0.018), higher levels of LDH (p < 0.01), lower levels of albumin (p < 0.01) and lower levels of hemoglobin (p < 0.01). The high LAR group also had a higher portion of colon cancer (p < 0.01), TNM stage II (p = 0.014) and tumor size > 5 cm (p < 0.01). More detailed information was shown in Table 1.

**Table 1:** Baseline information between the high LAR group and the low LAR group

Characteristics	Low LAR (3440)	High LAR (428)	P value^a^	P value^b^
Age, year	62.3 ± 12.1	67.9 ± 11.3	< 0.01*	
Sex				< 0.01*
Male	2065 (60.0%)	214 (50.0%)		
Female	1375 (40.0%)	214 (50.0%)		
BMI, kg/m2	22.8 ± 3.2	22.0 ± 3.4	< 0.01*	
Smoking	1332 (38.7%)	130 (30.4%)		< 0.01*
Drinking	1078 (31.3%)	106 (24.8%)		< 0.01*
Hypertension	878(25.5%)	132 (30.8%)		0.018*
T2DM	421 (12.2%)	58 (13.6%)		0.437
Neoadjuvant therapy	200 (5.8%)	32 (7.5%)		0.173
Lactate dehydrogenase, U/L	264.1 ± 119.0	630.7 ± 854.1	< 0.01*	
Albumin, g/L	40.9 ± 5.1	34.0 ± 5.9	< 0.01*	
Hemoglobin, g/L	123.0 ± 23.9	110.3 ± 23.9	< 0.01*	
LAR	6.6 ± 3.1	18.4 ± 21.8	< 0.01*	
Tumor location				< 0.01*
Colon	1560 (45.3%)	263 (61.4%)		
Rectum	1880 (54.7%)	165 (38.6%)		
TNM stage				0.014*
I	702 (20.4%)	62 (14.5%)		
II	1461 (42.5%)	191 (44.6%)		
III	1277 (37.1%)	175 (40.9%)		
Tumor size				< 0.01*
< 5 cm	2052 (59.7%)	199 (46.5%)		
≥ 5 cm	1388 (40.3%)	229 (53.5%)		

Note: Variables are expressed as the mean ± SD, n (%), ^a^ Calculated using independent sample t-test, ^b^ Calculated using the Chi-square test or Fisher’s exact test, *P-value < 0.05

Abbreviations: LAR, lactate dehydrogenase-to-albumin ratio; T2DM, type 2 diabetes mellitus; BMI, body mass index

### Short-term outcomes

Table 2 showed the Short-term outcomes between the two different groups. As a result, the surgery time was significantly longer in the high LAR group than in the low LAR group(p < 0.01), and the intro-operative blood loss was significantly more in the high LAR group(p < 0.01). The postoperative hospital stays were longer for the high LAR group (p < 0.01). Moreover, the high LAR group exhibited a higher incidence of overall complications (p < 0.01) and major complications (p < 0.01) compared to the low LAR group .

**Table 2:** Short-term outcomes between the high LAR group and the low LAR group

Characteristics	Low LAR (3440)	High LAR (428)	P value^a^	P value^b^
Operation time (minutes)	223.3 ± 77.9	234.4 ± 98.9	< 0.01*	
Blood loss (mL)	92.7 ± 122.7	124.0 ± 161.3	< 0.01*	
Hospital stay (days)	10.9 ± 8.8	12.4 ± 7.7		< 0.01*
Overall complications	693 (20.1%)	135 (31.5%)		< 0.01*
Major complications	70 (2.0%)	19 (4.4%)		< 0.01*

Note: Variables are expressed as the mean ± SD, n (%), ^a^ Calculated using independent sample t-test, ^b^ Calculated using the Chi-square test or Fisher’s exact test, *P-value < 0.05

Abbreviations: LAR, lactate dehydrogenase-to-albumin ratio

Univariate and multivariate analyses were conducted to find out the factors associated with overall complications. In univariate analysis, it was found that age [OR/95% CI: (1.024/1.018 to 1.031), p < 0.01], BMI [OR/95% CI: (0.970/0.947 to 0.994), p = 0.015], hypertension [OR/95% CI: (1.377/1.164 to 1.630), p < 0.01], T2DM [OR/95% CI: (1.602/1.293 to 1.985), p < 0.01], smoking [OR/95% CI: (1.230/1.051 to 1.438), p = 0.010], tumor size [OR/95% CI: (1.213/1.039 to 1.416), p = 0.014], hemoglobin [OR/95% CI: (0.992/0.989 to 0.995), p = 0.014], and LAR [OR/95% CI: (1.826/1.465 to 2.276), p < 0.01] were risk factors. Moreover, multivariate analysis showed that age [OR/95% CI: (1.017/1.010 to 1.024), p < 0.01], T2DM [OR/95% CI: (1.386/1.104 to 1.741), p < 0.01], smoking [OR/95% CI: (1.347/1.146 to 1.585), p < 0.01], hemoglobin [OR/95% CI: (0.995/0.991 to 0.998), p < 0.01], and LAR [OR/95% CI: (1.555/1.237 to 1.954), p < 0.01] were independent risk factors for overall complications (Table 3).

**Table 3:** Univariate and multivariate logistic regression analysis of the overall complications

Risk factors	Univariate logistic regression analysis		Multivariate logistic regression analysis	
	OR (95% CI)P value		OR (95% CI)P value	
Age, year	1.024 (1.018–1.031)	< 0.01*	1.017 (1.010–1.024)	< 0.01*
Sex (female/male)	0.862 (0.737–1.009)	0.065		
BMI, Kg/m2	0.970 (0.947–0.994)	0.015*	0.979 (0.954–1.004)	0.101
Hypertension (yes/no)	1.377 (1.164–1.630)	< 0.01*	1.158 (0.962–1.394)	0.121
T2DM (yes/no)	1.602 (1.293–1.985)	< 0.01*	1.386 (1.104–1.741)	< 0.01*
Tumor location (colon/ rectum)	0.962 (0.825–1.122)	0.624		
Tumor stage (III/II/I)	1.037 (0.934–1.151)	0.498		
Smoking (yes/no)	1.230 (1.051–1.438)	0.010*	1.347 (1.146–1.585)	< 0.01*
Drinking (yes/no)	1.026 (0.869–1.211)	0.763		
Tumor size (≥ 5/<5), cm	1.213 (1.039–1.416)	0.014*	1.085 (0.923–1.274)	0.324
Hemoglobin, g/L	0.992 (0.989–0.995)	0.014*	0.995 (0.991–0.998)	< 0.01*
LAR (high/low)	1.826 (1.465–2.276)	< 0.01*	1.555 (1.237–1.954)	< 0.01*

Note: *P-value < 0.05.

Abbreviations: OR, Odds ratio; CI, confidence interval; BMI, body mass index; T2DM, type 2 diabetes mellitus; LAR, lactate dehydrogenase-to-albumin ratio

As for major complications, it was found that age [OR/95% CI: (1.028/1.010 to 1.048), p < 0.01], tumor location [OR/95% CI: (0.563/0.361 to 0.878), p = 0.011], and LAR [OR/95% CI: (2.236/1.333 to 3.752), p < 0.01] were risk factors in univariate analysis. Furthermore, age [OR/95% CI: (1.027/1.007 to 1.046), p < 0.01], tumor location [OR/95% CI: (0.503/0.321 to 0.790), p < 0.01], and LAR [OR/95% CI: (2.178/1.279 to 3.707), p < 0.01] were independent predictors for major complications in multivariate analysis. (Table 4).

**Table 4:** Univariate and multivariate logistic regression analysis of the major complications

Risk factors	Univariate logistic regression analysis		Multivariate logistic regression analysis	
	OR (95% CI)P value		OR (95% CI)P value	
Age, year	1.028 (1.010–1.048)	< 0.01*	1.027 (1.007–1.046)	< 0.01*
Sex (female/male)	0.464 (0.285–0.754)	0.065		
BMI, Kg/m2	1.024 (0.960–1.093)	0.470		
Hypertension (yes/no)	1.452 (0.930–2.267)	0.101		
T2DM (yes/no)	1.448 (0.824–2.544)	0.198		
Tumor location (colon/ rectum)	0.563 (0.361–0.878)	0.011*	0.503 (0.321–0.790)	< 0.01*
Tumor stage (III/II/I)	0.903 (0.681–1.199)	0.482		
Smoking (yes/no)	1.417 (0.929–2.161)	0.105		
Drinking (yes/no)	1.484 (0.964–2.283)	0.073		
Tumor size (≥ 5/<5), cm	1.088 (0.712–1.662)	0.607		
Hemoglobin, g/L	1.005 (0.996–1.014)	0.279		
LAR (high/low)	2.236 (1.333–3.752)	< 0.01*	2.178 (1.279–3.707)	< 0.01*

Note: *P-value < 0.05.

Abbreviations: OR, Odds ratio; CI, confidence interval; BMI, body mass index; T2DM, type 2 diabetes mellitus; LAR, lactate dehydrogenase-to-albumin ratio

### Univariate and multivariate analysis of OS

We also performed Cox regression analyses to detect independent predictors for OS. In univariate analysis, we found that age [HR/95% CI: (1.047/1.038 to 1.055), p < 0.01], BMI [HR/95% CI: (0.953/0.926 to 0.981), p < 0.01], tumor stage [HR/95% CI: (2.133/1.853 to 2.455), p < 0.01], tumor size [HR/95% CI: (1.494/1.252 to 1.784), p < 0.01], hemoglobin [HR/95% CI: (0.993/0.989 to 0.996), p < 0.01], and LAR [HR/95% CI: (2.476/2.009 to 3.053), p < 0.01] were risk factors. Moreover, age [HR/95% CI: (1.041/1.032 to 1.049), p < 0.01], tumor stage [HR/95% CI: (2.079/1.802 to 2.398), p < 0.01], tumor size [HR/95% CI: (1.260/1.050 to 1.513), p = 0.013] as well as LAR [HR/95% CI: (1.930/1.554 to 2.398), p < 0.01] were independent predictors for OS in multivariate analysis. However, BMI [HR/95% CI: (0.986/0.958 to 1.014), p = 0.318], and hemoglobin [HR/95% CI: (1.001/0.997 to 1.005), p = 0.781] were not independent predictors (Table 5).

**Table 5:** Univariate and multivariate analysis of overall survival

Risk factors	Univariate analysis			Multivariate analysis	
	HR (95% CI)		P value	HR (95% CI)P value	
Age, year	1.047 (1.038–1.055)	< 0.01*	1.041 (1.032–1.049)		< 0.01*
Sex (female/male)	0.861 (0.718–1.033)	0.108			
BMI, Kg/m2	0.953 (0.926–0.981)	< 0.01*	0.986 (0.958–1.014)		0.318
Hypertension (yes/no)	0.961 (0.782–1.181)	0.707			
T2DM (yes/no)	1.261 (0.976–1.629)	0.076			
Tumor location (colon/ rectum)	1.170 (0.981–1.397)	0.082			
Tumor stage (III/II/I)	2.133 (1.853–2.455)	< 0.01*	2.079 (1.802–2.398)		< 0.01*
Smoking (yes/no)	1.068 (0.891–1.280)	0.478			
Drinking (yes/no)	1.032 (0.852–1.250)	0.747			
Tumor size (≥ 5/<5), cm	1.494 (1.252–1.784)	< 0.01*	1.260 (1.050–1.513)		0.013*
Hemoglobin, g/L	0.993 (0.989–0.996)	< 0.01*	1.001 (0.997–1.005)		0.781
LAR (high/low)	2.476 (2.009–3.053)	< 0.01*	1.930 (1.554–2.398)		< 0.01*

Note: *P-value < 0.05.

Abbreviations: HR, hazard ratio; CI, confidence interval; BMI, body mass index; T2DM, type 2 diabetes mellitus, LAR, lactate dehydrogenase-to-albumin ratio

### Univariate and multivariate analysis of DFS

In terms of DFS, we also found that age [HR/95% CI: (1.033/1.026 to 1.040), p < 0.01], BMI [HR/95% CI: (0.974/0.950 to 0.999), p = 0.040], tumor stage [HR/95% CI: (2.037/1.799 to 2.305), p < 0.01], tumor size [HR/95% CI: (1.335/1.139 to 1.564), p < 0.01], hemoglobin [HR/95% CI: (0.994/0.991 to 0.998), p < 0.01], and LAR [HR/95% CI: (2.094/1.720 to 2.551), p < 0.01] were risk factors in univariate analysis. Then, in multivariate analysis, age [HR/95% CI: (1.029/1.021 to 1.036), p < 0.01], tumor stage [HR/95% CI: (2.004/1.768 to 2.272), p < 0.01], and LAR [HR/95% CI: (1.750/1.427 to 2.146), p < 0.01] were independent predictors for DFS. But BMI [HR/95% CI: (0.998/0.973 to 1.023), p = 0.866], tumor size [HR/95% CI: (1.151/0.978 to 1.354), p = 0.092], and hemoglobin [HR/95% CI: (1.000/0.997 to 1.004), p = 0.793] were not independent predictors (Table 6).

**Table 6:** Univariate and multivariate analysis of disease-free survival

Risk factors	Univariate analysis			Multivariate analysis	
	HR (95% CI)		P value	HR (95% CI)P value	
Age, year	1.033 (1.026–1.040)	< 0.01*	1.029 (1.021–1.036)		< 0.01*
Sex (female/male)	0.864 (0.734–1.017)	0.078			
BMI, Kg/m2	0.974 (0.950–0.999)	0.040*	0.998 (0.973–1.023)		0.866
Hypertension (yes/no)	0.983 (0.819–1.180)	0.854			
T2DM (yes/no)	1.133 (0.878–1.412)	0.377			
Tumor location (colon/ rectum)	1.098 (0.937–1.286)	0.249			
Tumor stage (III/II/I)	2.037 (1.799–2.305)	< 0.01*	2.004 (1.768–2.272)		< 0.01*
Smoking (yes/no)	1.095 (0.931–1.286)	0.273			
Drinking (yes/no)	1.042 (0.878–1.236)	0.638			
Tumor size (≥ 5/<5), cm	1.335 (1.139–1.564)	< 0.01*	1.151 (0.978–1.354)		0.092
Hemoglobin, g/L	0.994 (0.991–0.998)	< 0.01*	1.000 (0.997–1.004)		0.793
LAR (high/low)	2.094 (1.720–2.551)	< 0.01*	1.750 (1.427–2.146)		< 0.01*

Note: *P-value < 0.05.

Abbreviations: HR, hazard ratio; CI, confidence interval; BMI, body mass index; T2DM, type 2 diabetes mellitus, LAR, lactate dehydrogenase-to-albumin ratio

### OS/ DFS on different tumor stages

**Fig. 2:**
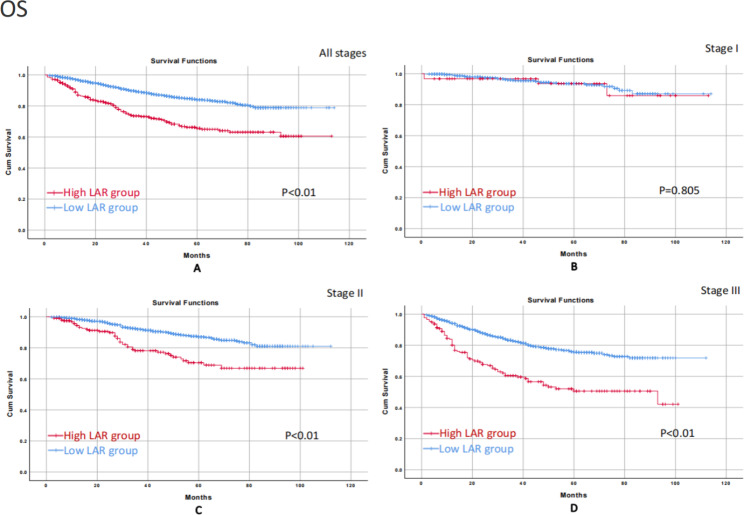
Kaplan-Meier survival curve for the impact of LAR on OS of patients in all stages and stage I-III.

**Fig. 3:**
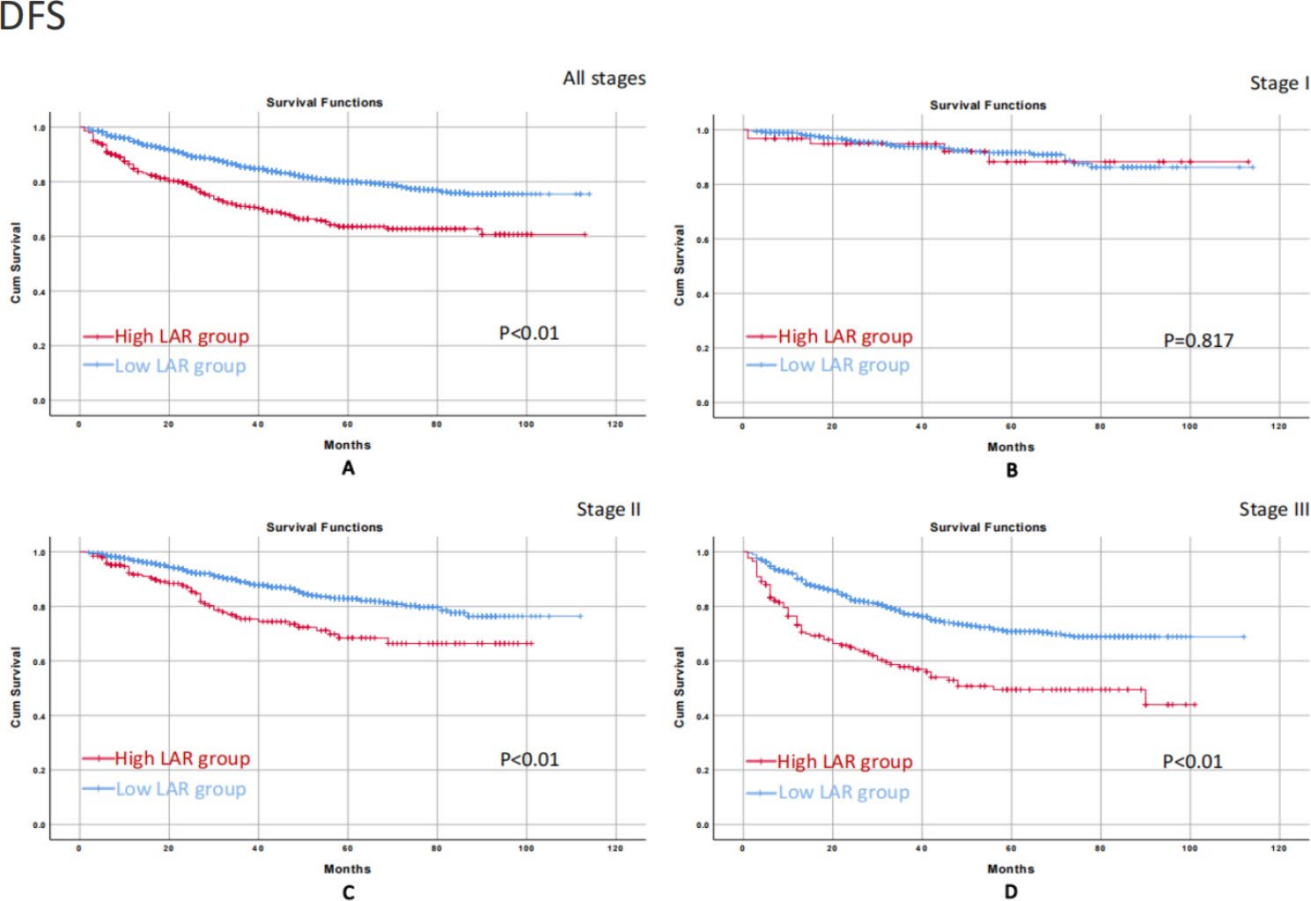
Kaplan-Meier survival curve for the impact of LAR on DFS of patients in all stages and stage I-III.

The median follow-up time was 34(1-114) months. We conducted the Kaplan-Meier survival curve to further evaluate the impact of LAR on prognosis at the different tumor stages. In terms of OS, the high LAR group correlated with worse OS in all stages (p < 0.01), stage II (p < 0.01), and stage III (p < 0.01) than the low LAR group. No significant difference was found in stage I (p = 0.805) (Fig. 2). As for DFS, the high LAR group associated with worse DFS in all stages (p < 0.01), stage II (p < 0.01), and stage III (p < 0.01) than the low LAR group. No significant difference was found in stage I (p = 0.817) (Fig. 3).

## Discussion

In this current study, higher LAR exhibited longer operation time, larger intro-operative blood loss, extended postoperative hospital stays, and a higher incidence of overall complications and major complications. Moreover, LAR was established as an independent risk factor for overall complications and major complications. In terms of survival, higher LAR correlated with worse OS and DFS in all stages, stage II and III. Furthermore, LAR was identified as an independent predictor for OS and DFS.

Previous studies had demonstrated that elevated LDH and decreased albumin were correlated with poor survivals in many kinds of cancers [23–27]. LDH, as one of the glycolytic enzymes, could convert pyruvate to lactate and create a micro-hypoxic environment, which led to tumor hypoxia, neo-angiogenesis, and poor prognosis of various tumors [28]. LAR, combing LDH and albumin, was more effective than each alone. Peng et al. [29] made a nomogram based on LAR and PLR to predict survival in nasopharyngeal carcinoma and they revealed that LAR served as an independent prognostic risk factor for OS and DFS. Nakazawa et al. [30] found that LAR was correlated with the sensitivity and therapeutic resistance of Nivolumab in patients with gastric cancer, which might be helpful to the immunotherapies. As for CRC, Aday et al. [20] included 295 CRC patients who underwent curative resection and got a result that high pretreatment LAR level was an unfavorable prognosticator. Their number of cases was relatively small. Hu et al. [19] did a comparison between the LAR and the TNM staging system, and they identified that LAR could be served as a reliable prognostic factor for OS and DFS. Moreover, Wu et al. [31] conducted a retrospective study and found that the levels of LAR were correlated with T stage and TNM stage of CRC patients. They reported that patients diagnosed with T4 stage or IV stage had higher LAR, suggesting that higher LAR was associated with poor prognosis in CRC patients. However, none of them demonstrated the relationship between LAR and short-term outcomes. From what we know, this study was firstly explore the effect of LAR on the short-term outcomes including overall and major complications of CRC patients after radical resection. And our samples were by far the largest.

In our study, we defined the high LAR group as LAR > 12.3 and the low LAR group was defined as LAR ≤ 12.3 according to the X-tile software. Then, we investigated the correlation between LAR and short-term outcomes. We found that high LAR correlated with more postoperative complications. The serum albumin, as an indicator of the nutritional status of the body, was associated with system inflammation [32, 33]. The decreased albumin had huge influence on recovery after surgery [34, 35], which might lead to severe postoperative complication [4, 36, 37]. LDH was involved in tumor metabolism [38, 39], and elevated LDH was associated with immune suppression [40]. Therefore, high LDH might play an important role in promoting tumors’ progression. We found that LAR was associated with long-term prognosis including OS and DFS, and finally, our Kaplan-Meier survival curve analysis confirmed that LAR served as a prognostic indicator for OS and DFS across all tumor stages, as well as in stage II and III, which might catch surgeon’s attention for earlier postoperative chemoradiotherapies and more frequent follow-up reviews [41, 42].

Several limitations were present in this study. First, being a single-center retrospective study, the findings may have limited generalizability beyond the specific regions covered in this investigation. Second, the sample size of patients in stage I was relatively small, which might lead to data bias. Third, the lack of information on postoperative chemoradiotherapies could potentially affect the robustness of the survival analysis, representing an additional study limitation. Here in, more detailed and multi-center prospective randomized controlled trials studies were needed for further exploration.

## Conclusion

Elevated LAR was linked to increased postoperative complications, as well as poorer overall survival (OS) and disease-free survival (DFS) among CRC patients who underwent radical resection. Additionally, LAR emerged as an independent predictor for overall complications, major complications, OS, and DFS. These findings underscore the importance of surgeons giving careful consideration to LAR when making clinical decisions.

## Data Availability

The data are available from the corresponding author on reasonable request.
